# Beyond the Categorical Distinction Between Borderline Personality Disorder and Bipolar II Disorder Through the Identification of Personality Traits Profiles

**DOI:** 10.3389/fpsyt.2020.00552

**Published:** 2020-08-14

**Authors:** Juana Villarroel, Valeria Salinas, Hernán Silva, Luisa Herrera, Cristián Montes, Sonia Jerez, Paul A. Vöhringer, Maria Leonor Bustamante

**Affiliations:** ^1^ University Psychiatric Clinic, Faculty of Medicine, Universidad de Chile, Santiago, Chile; ^2^ Neurogenetics Outpatient Clinic and Laboratory, University Neurology Center and Neurology Section, J.M. Ramos Mejía Hospital, Faculty of Medicine, Universidad de Buenos Aires, Buenos Aires, Argentina; ^3^ Precision Medicine and Clinical Genomics Program, Faculty of Biomedical Sciences, Translational Medicine Research Institute, Universidad Austral-CONICET, Buenos Aires, Argentina; ^4^ Human Genetics Program, Faculty of Medicine, Biomedical Sciences Institute, Universidad de Chile, Santiago, Chile; ^5^ Mood Disorders Program, Tufts Medical Center, Tufts University, Boston, MA, United States

**Keywords:** five-factor model, neuroticism, agreeableness, conscientiousness, transdiagnostic approach

## Abstract

**Background:**

The relationship between borderline personality disorder (BPD) and type-II bipolar disorder (BDII) is not clearly understood. Nevertheless, in clinical practice and research, most efforts focus on establishing a categorical distinction between the two. We propose using personality traits as a more informative strategy to describe them.

**Methods:**

Five-Factor Model personality traits were measured in 73 individuals with either BPD or BDII. Latent class cluster analysis was applied to the sample.

**Results:**

A three-cluster model resulted the best fit to the data, where all clusters had high neuroticism and low extraversion scores but differed widely on the other traits. The clusters’ boundaries did not match the categorical diagnosis.

**Conclusions:**

Our sample showed significant heterogeneity on personality traits, which can have a relevant effect on the outcome of each disorder and that was not captured by the categorical diagnosis. Thus, we advocate for a multivariate approach as a better way to understand the relationship between BPD and BDII.

## Introduction

The distinction between borderline personality disorder (BPD) and bipolar disorder (BD) has been traditionally considered a major challenge for clinicians and researchers alike (1). Altogether, there is sufficient evidence to sustain that BPD and BD can be considered separate entities (2). However, it is also clear that the relationship between them is complex, with frequent comorbidity, overlapping clinical definitions, and possibly common risk factors (3). The majority of the studies agree that both disorders coexist much more frequently than expected by chance, in an average 1/5 of the patients, however whether this is due to misdiagnosis or to a common underlying biology is not clear (4). However difficult it is to establish a categorical distinction, this is the key element used to select the best treatment. Therefore, research has focused in looking for specific markers to establish a diagnosis of either BD or BPD. An episodic pattern of symptoms, the presence of hyperactivity, and a positive family history are all suggestive of BD, whereas a personal history of sexual abuse suggests BPD (5–7). However, although these features support a diagnosis when they are present, they do not rule it out if absent, which is a problem when analyzing any disorder that presents heterogeneity. For instance, di Giacomo et al. (8) found that a clear distinction between BPD and BD in a mixed or manic state could be established based on the pattern of mood symptoms. On the other hand, patients with type-II bipolar disorder (BDII)do not experience manic episodes. Thus, in patients with a history of milder symptoms, it will be difficult for patients, relatives, and clinicians to attribute the symptoms either to a self-limited mood episode (as in BDII) or to persistent affective lability and increased sensitivity to environmental stressors (typical of BPD). Furthermore, the diagnostic dichotomy may not capture all the aspects that are relevant for assessing long-term outcome. Because there is significant heterogeneity among each of these disorders, other specific features that influence the outcome like impulsivity, or temperament, cannot be directly inferred from the category (9). Moreover, in the case of comorbidity, the effect one disorder on the other appears to be asymmetrical, as it has been observed that the presence of BPD worsens the outcome of BDII but not vice versa; the reasons for this asymmetry are not clear (4). In sum, this is a prime example of the long-standing challenges faced by psychiatric nosology as a discipline focused in defining valid disease entities with solid neurobiological correlates and reliable diagnostic tools. According to Stoyanov et al. (10), although most psychiatric diagnosis cannot fulfill the concept of validity in a whole sense, they can be useful because of the information they provide on outcome, treatment response, and etiology. This holds for BPD and BDII, and in fact this distinction has led to defining and testing treatment guidelines. However, because evidence-based guidelines have shown major limitations in efficacy, there is growing recognition of the need to move to “personalized” or “precision” practice. This will require identifying the sources of interindividual variation that can challenge the predictions based on the average response of one diagnostic category (11).

Among the clinical variation that have a relevant role on long-term outcome are personality traits. Although there are several proposals, including some with solid empirical foundations, we have chosen the Five-Factor model (12–14) as our approach because of its extensive validation. It describes personality in terms of five dimensions: neuroticism (N), extraversion (E), agreeableness (A), openness to experience (O), and conscientiousness (C). This model has been extensively replicated in several clinical and non-clinical samples. It is commonly accepted that BPD is characterized by (maladaptive) high levels of N, whereas BD has high levels of the E. However, there is not enough evidence supporting this notion, specially regarding the milder form of BD. Thus, to contribute to understand the relationship between BPD and BDII, we aimed to verify whether each of these disorders presented differential profiles of personality traits. We studied a sample of patients with either BPD or BDII using Latent Class Cluster Analysis (LCA). This method is used to look for substructures within groups of individuals that are not determined by the observed variables but for other variables (“latent”) that can be inferred from the former. In this case, if BPD and BD have clearly distinct profiles, our analysis would verify that our sample was composed by two subgroups, each composed only by individuals with one and the same diagnosis. On the other hand, finding anything different from subgroups matching the original categories would indicate that the categorical distinction does not fully capture the intra-diagnosis heterogeneity and favor a transdiagnostic approach instead.

## Materials and Methods

### Subjects

Seventy-three patients (43 with BPD, and 30 with BDII) were recruited at the University Psychiatric Clinic of Universidad de Chile. The Ethics Committee of the Faculty of Medicine of Universidad de Chile approved the protocol, and all individuals signed an informed consent form in accordance with the Declaration of Helsinki. The University Psychiatric Clinic is a referral center for the Metropolitan Area in Chile, that provides specialist care for patients that due to the severity of their illness cannot be managed by primary care. It has separate units for adults, and children and adolescents. The Adult section is organized in specialized units focused on specific problems (bipolar disorders, substance use disorders, psychosis, personality disorders, and general psychiatry) where they receive in- or out-patient treatment according to their needs. All patients with a diagnosed psychiatric disorder receive pharmacological treatment, according to national guidelines (if available) or international consensus guidelines. The present study was carried out by the Personality Disorders Unit, and recruited individuals that either entered the Unit directly or that were referred to the study from other Unit by their attending physician. All individuals had to be 18 or older to enter the study. All individuals were interviewed either by a psychiatrist or by a senior year Psychiatry resident who carried out a semi-structured interview and applied the structured interviews. As part of the evaluation, all patients were discussed separately with two senior psychiatrist from the research team (HS and SJ), who have extensive clinical and academic experience. Where there was no agreement on the diagnosis of BPD or BDII between the two senior psychiatrists, the individual was excluded from the study. Co-existence of BPD and BDII diagnoses was an exclusion criterion. Other exclusion criteria were history of mania or psychosis (*i.e.*, any indicators of type-I BD), present substance abuse, present depressive episode. Medical illness that could mimic or exacerbate a mental disorder (e.g., metabolic disorders, neurological disorders), as established by anamnesis, was also an exclusion criterion.

### Procedures

#### Clinical Evaluations

Patients underwent an evaluation including the Structured Clinical Interview for DSM-IV Axis (SCID-I) (15), as well as either the International Personality Disorders Examination (IPDE) (16) or the Structured Clinical Interview for DSM-IV Axis II Disorders (SCID-II) (17).

Personality traits from the Five-Factor Model (FFM) [i.e., neuroticism (N), extraversion (E), Agreeableness (A), openness to experience (O), and conscientiousness (C)] were measured using the Revised Neo Personality Inventory (NEO-PiR), Spanish-validated version (18).

#### Statistical Analysis

Univariate statistical analysis was performed using SPSS 13.0. Descriptive statistics included estimation of means and SD. Shapiro-Wilk test was used to establish normality of the data. Between-groups comparisons were made through one-tailed Student´s-T test or Kruskal-Wallis test.

Latent class cluster analysis, (LCA (19) was using Latent Gold 11.0. The variables studied were the T-scores on the 5 personality traits of the FFM, using diagnosis, age, and gender were used as covariates. Models including 1 to 10 classes were fitted to the data, and the best model was selected according to Bayesian Information Criterion, BIC.

## Results

The sample was composed of 73 patients, 43 with BPD 30 with BDII. 71% of the sample were female. There were no significant differences in female/male distribution between BPD and BDII groups (*p*=0.295).

**Figure 1:**
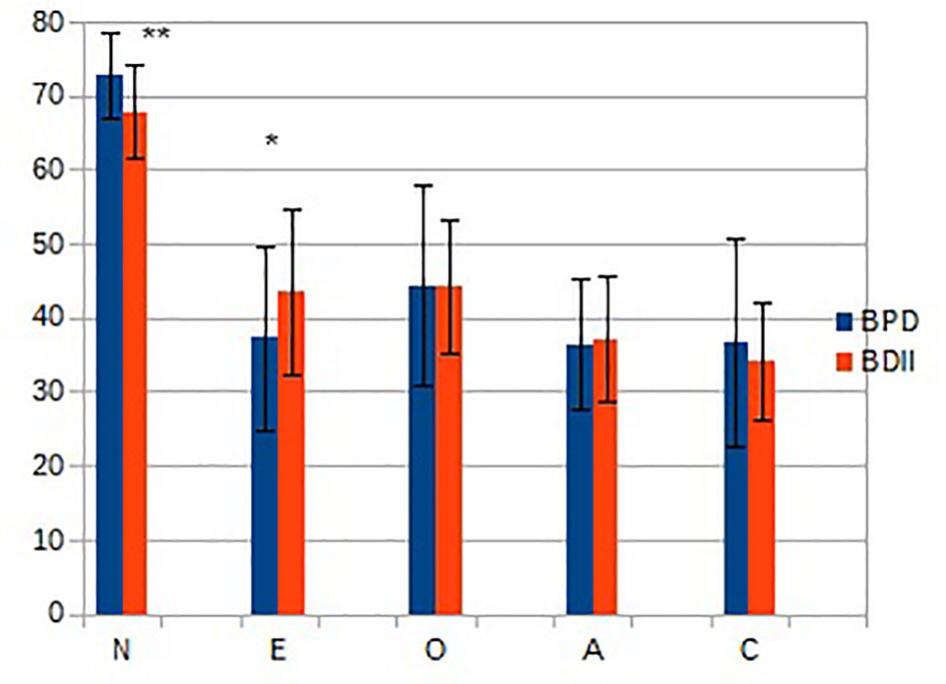
Personality traits scores (mean ± SD) in the two groups of patients. BPD, Borderline Personality Disorder; BDII, type-II Bipolar Disorder. *p < 0.05; **p < 0.01. N, neuroticism; E, extraversion; O, openness; A, agreeableness; C, conscientiousness.

**Figure 2:**
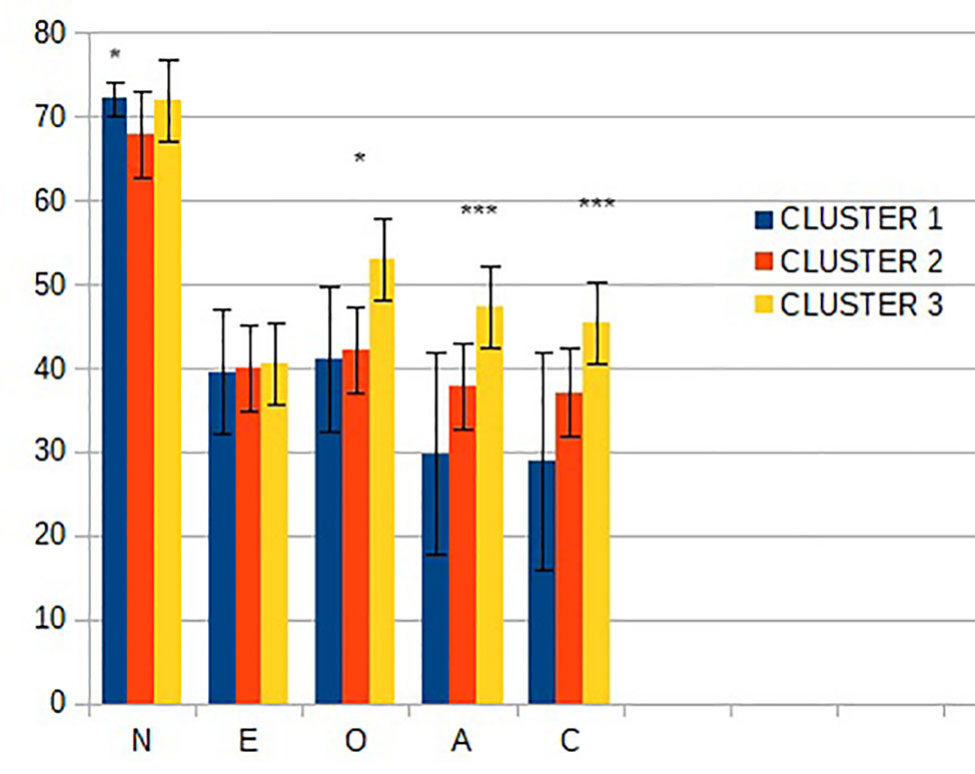
Personality traits scores (mean ± SD) in the three subgroups defined by Latent Class Cluster Analysis. N, neuroticism; E, extraversion; O, openness; A, agreeableness; C, conscientiousness; *p < 0.05; ***p < 0.001.

**Figure 1** depicts personality traits scores (mean ± SD) as measured by NEO PI-R in the two groups of patients. There are statistically significant differences on N and E. While the two groups have high N and low E compared to general population, the scores are more extreme in BPD. Furthermore, LCA (depicted in **Figure 2**) identified three subgroups (“clusters”) defined by personality traits. All three had very high scores of N and low E. The profile for the other traits can be summarized as follows: Cluster 1: low O, very-low A/C; Cluster 2: low O/A/C; Cluster 3: mean O/A/C.

Patients with each diagnosis were indistinctly distributed among the subgroups, as follows. For BDP, 44,1% of all the patients belonged to Cluster 1; 25.5% to Cluster 2; and 30,2% to Cluster 3; for BDII, 40% belonged to Cluster 1; 43% to Cluster 2; and 16.7% to Cluster 3. Thus, there was no coincidence between a categorical diagnosis and one specific personality profile.

## Discussion

Our results provide new insight into the implications of the distinction between BPD and BDII. A vast amount of research has focused on defining clinical variables that can correctly classify patients with BPD from those with BDII, and, currently, this discrimination has a major impact on treatment, because clinical guidelines have been developed separately, and patients are treated in different specialized Units in several centers (including our own University Hospital). According to our own data and the literature, this distinction is, in fact, possible and justified by objective measures. However, from our observations we also conclude that it can be largely uninformative. Our sample was composed of individuals reliably classified as having either BDII or BPD, using structured interviews and expert inference. Inconclusive cases were excluded, as were those where the two disorders were present concomitantly. Thus, we are fully confident that we have successfully sorted out the individuals in our sample. Then, while we observe a statistically significant difference on N and E between BPD and BDII, we also acknowledge that the N and E levels from the whole sample fall out of the normal range. Therefore, we do not expect that this observation will be useful in clinical decision making. We observed that there is extensive heterogeneity inside each group, and as the subgroups defined by LCCA are composed by a mixture of individuals with BPD or BDII, we rule out that there is a characteristic personality traits profile for either disorder. We consider that these newly defined subgroups may be clinically more relevant, because personality traits have a significant influence on long-term outcome, and thus we propose that in clinical and research settings, it is imperative to go beyond the categorical distinction and purposefully characterize other relevant variables.

Our sample is consistent with other clinical samples, characterized by high scores on N and low on all other traits (20, 21). When comparing between the categorically defined groups, we observed significant differences in N and E between these two groups, with less extreme scores on BDII. Other groups have also observed significant, albeit subtle, differences in clinical traits between these two groups, e.g., impulsivity, mood instability, and emotional dysregulation (22–24). Based on the hypothesis that the categorical distinction could be failing to account for intra-diagnosis heterogeneity, we then sought to verify whether there was a specific profile of symptoms for each disorder. Instead we were able to distinguish three profiles, which did not respect the boundaries of the categorical diagnosis.

The different profiles have potential clinical implications. Personality traits have shown to predict relevant outcomes in clinical and population samples. For instance, subjects with higher N are more vulnerable to stress and have more emotional reactivity, are often shy and impulsive, and have deficient interpersonal skills. Therefore, these individuals are at an elevated risk of having mental disorders or having a more severe form of a mental disorder (21). A recent study in type-I bipolar disorder identified two N-facets as relevant predictors for suicide, albeit with a smaller effect than mood disturbance (25). Moreover, Su et al. (26) found that high levels of N, particularly when associated to low levels of E were predictors of suicidal behavior in individuals with no history of mood disorder. In our sample, although N is significant in separating the clusters, all three of them score very high in comparison to the general population. Thus, our sample appears to be uniformly influenced by this risk factor. On the other hand, larger differences are observed between subgroups in the levels of A, O, and C. It is possible that this can have an impact on the outcome, including the likelihood to seek medical care, maintain healthy habits, and establish an active social role social network. The protective role of A, O, and C in non-clinical populations has been consistently established. From that, one might simplistically expect that cluster 1 displays a worse outcome, having the lowest levels of these three traits. However, the literature considering clinical samples is less abundant, and recent evidence suggests that results from community samples cannot be readily translated to clinical settings. In fact, a study (20) found that non-suicidal self-injury and impulsive behaviors in BPD patients were predicted by higher levels of N, E, A, and C. As the authors hint, this apparently counter-intuitive finding may be mediated by other characteristics of BDP functioning, like emotional regulation, stress coping mechanisms, and interpersonal problems. Personality traits appear not to exert a direct effect but interact with the disorder core symptoms to modify the clinical outcome. Therefore, we do not propose eliminating the categorical distinction altogether, but rather considering a multifaceted approach. Thus, resources, like psychotherapy and psychoeducation, that work on modulate personality traits can be better aligned with the primary treatment. In our current practice, much effort is allocated to sort out patients with BD from BPD. After this initial step, patients enter one of two separate track, each with its own treatment recommendations. It is often the case that BD guidelines are mainly focused on optimizing pharmacological therapy while in BPD units the emphasis goes on psychotherapy. In some centers, including our own University Hospital, this takes place in specific treatment Units. Moreover, training psychiatrists learn to treat patients after they have been sorted out. Our results underscore that interindividual variation is not best captured by this categorical distinction, and therefore, their management should not be solely based on it.

The main strengths of our study are focusing on type II-BD, which is especially challenging due to its milder symptoms and has received less attention from researchers; and having excluded the confounding effect of comorbidity in our sample. However, we must also acknowledge relevant limitations. BPD patients were recruited mostly in an outpatient treatment setting, and thus do not represent the whole range of severity of BPD and its high rate of hospitalization. In addition, whereas our patients were currently free from depression and had no history of mania, we did not use a mania scale at the time of the study, and thus we cannot rule out subsyndromal mood variations that could alter the measurements, especially among BDII. Furthermore, we did not get follow-up information on the subjects of our study as to establish a clear prognostic value of these variables in our sample.

In spite of these limitations, in light of our results and previous literature we advocate for a diagnostic approach that goes beyond the categorical distinction, and uses dimensional information for a better understanding of the implications of BPD, BDII, and their combination.

## Data Availability Statement

The datasets generated for this study are available on request to the corresponding author.

## Ethics Statement

The study was reviewed and approved by Ethics Committee for Human Subjects Research Faculty of Medicine, University of Chile. The patients/participants provided their written informed consent to participate in this study.

## Author Contributions

JV: Conceived the analysis, collected data, provided critical input for the manuscript. VS: Performed the analysis, wrote the first draft. HS: Conceived the analysis, provided critical input for the manuscript. LH: Designed the analysis, provided critical input for the manuscript. SJ: Conceived the analysis, provided critical input for the manuscript. CM: Collected data, provided critical input for the manuscript. PV: Collected data, Provided critical input for the manuscript. MB: Conceived and designed the analysis, led the writing of the manuscript with input from all authors.

## Funding

This work was funded by FONDECYT, CONICYT, Chilean Secretary of Education, grant numbers 1030305, 1071045 and by Líneas de Apoyo a la Investigación financiadas por ICBM 2020 Program. The funding agencies was not involved in collecting, analyzing, or interpreting the data.

## Conflict of Interest

The authors declare that the research was conducted in the absence of any commercial or financial relationships that could be construed as a potential conflict of interest.
